# Mental health of young informal carers: a systematic review

**DOI:** 10.1007/s00127-022-02333-8

**Published:** 2022-07-07

**Authors:** Ludmila Fleitas Alfonzo, Ankur Singh, George Disney, Jennifer Ervin, Tania King

**Affiliations:** 1 Disability and Health Unit, Centre for Health Equity, Melbourne School of Population and Global Health, The University of Melbourne, Parkville, Level 4, 207, Bouverie Street, 3010 Australia; 2 Centre of Epidemiology and Biostatistics, Melbourne School of Population and Global Health, The University of Melbourne, Parkville. Victoria, Australia

**Keywords:** Child, Adolescent, Youth, Caregiver, Mental health

## Abstract

**Purpose:**

This systematic review aims to assess and evaluate quantitative evidence on the association between informal caregiving and mental health in young people.

**Methods:**

This review was registered in PROSPERO (CRD42021251666). We conducted our search in the following four databases: Medline (PubMed and OVID), EMBASE, PsycInfo and Web of Science. The last search was performed on the 17th of March of 2021. Quantitative studies that focused on carers aged 25 years or less and compared the mental health status of carers and non-carers were eligible for inclusion. Two reviewers independently assessed articles for eligibility and performed the quality assessment using the Risk of Bias tool in Non-Randomised Studies of Exposures (ROBINS-E).

**Results:**

We identified a total of ten eligible articles. Mental health outcomes included depression, anxiety and other mental or emotional problems. Nine out of the ten studies showed that being a young carer was consistently associated with poor mental health. However, the overall quality of evidence was low, and longitudinal data were limited to three articles. The primary sources of bias were confounding and outcome measurement.

**Conclusion:**

Young carers experience poorer mental health outcomes than their non-caring peers. However, we identified an overall lack of quantitative evidence of high methodological rigour. To establish if young caring leads to poor mental health, future research should focus on addressing the identified methodological limitations and understanding the mechanisms explaining these associations. Addressing these gaps can better inform the allocation of appropriate support and resources to optimise the mental health of young carers.

**Supplementary Information:**

The online version contains supplementary material available at 10.1007/s00127-022-02333-8.

## Introduction

Globally, about 2–8% of people aged under 25 years provide regular unpaid support to someone with a disability, chronic condition, mental or alcohol/substance use problem or an elderly relative [1, 2]. Young informal carers play a vital role in the support of their caring recipient, taking on roles that would usually be associated with older adults [3–8]. These arrangements are highly demanding and often extremely time-consuming, placing young carers at risk of poor educational, economic and health outcomes [4, 9–11].

A substantial body of qualitative literature has been important in raising awareness and understanding of young carers [10–13]. Evidence suggests that young caring limits school participation and employment [10, 11]. To meet their caring demands, young carers are likely to skip school, arrive late or need to leave early [11]. Many feel overwhelmed and report difficulties balancing their school activities with caring demands [10, 11, 13]. Similar barriers are also reported for young carers who participate in the paid labour force [12, 13]. Moreover, the multiple demands imposed on young carers can restrict their social and leisure activities [14].

Young carers undertake different types of caring roles that are based on the needs of their caring recipients [7, 15]. As such, informal caregivers can be placed on a continuum depending on the type and amount of care provided [7]. Type of care may include support with core (mobility, communication and personal care) and non-core activities (transport and household chores) [15]. Many young carers who provide support with core activities spend a substantial amount of time on their caring roles. The provision of informal care can act as a chronic stressor, placing young carers at risk of developing mental disorders such as depression and anxiety [7]. Moreover, caring for parents or family members with severe chronic conditions may require complex medical support that young caregivers often shoulder with little or no training [16]. In turn, meeting these complex caring demands may exacerbate mental distress among young informal carers [16]

A large body of evidence has linked young caring with negative mental health outcomes [3–5, 9, 12, 17–21]. However, this evidence is not always consistent. While some authors report a high proportion of emotional problems [5], depression and anxiety in young carers [18]; others describe young carers reporting positive rather than negative mental health effects from their caregiving, with only a small subset displaying clinical levels of depression [20]. This lack of clarity in evidence hampers efforts to support and address the mental health needs of young people who provide informal care. This is a crucial gap to address given that mental health in adolescence and young adulthood commonly predicts future mental health [22–24].

The lack of clarity in evidence may be related to the absence of comparison groups and limited external validity of the current literature. Comparison groups are necessary to determine whether the mental health status of carers differs from that of non-carers, thereby helping identify the needs of young carers. Moreover, most previous studies use a convenience sampling strategy, recruiting young carers from organisations, making it difficult to generalise these findings to the wider population. This sampling strategy can also introduce selection bias where, for example, young carers with greater caring demands and less time to engage with these organisations are less likely to participate. Additionally, the sample size for many studies is small, further limiting the validity of these findings. These limitations have been highlighted in a recent literature review, where the authors called for large scale studies using representative samples and comparing outcomes of young carers and non-carers [25].

While large population-based studies using comparison groups in this research field are scant [25, 26], new research is emerging that utilises more representative sampling strategies and clear comparisons groups [27–29]. But once again, the evidence remains unclear. For example, a study using data of higher education students in Norway found that female and male young carers were more likely to display symptoms of anxiety and depression than their non-caring peers. Similar findings were reported for young carers in Scotland [30]. However, this was not the case for Australian students, where the distribution of anxiety and depression was reported to be similar for caring and non-caring youth [31].

To understand the impact of informal caring on youth mental health, it is important to assess the extent to which caring may be causally associated with mental health. Kavanaugh et al. [32] conducted a scoping review to map young carers, their caring roles and the impact of informal caring in US youth. They found a scarce body of quantitative research, with a lack of longitudinal research assessing the causal effects of young caring on later life outcomes and possible mediators for this relationship. A recent literature review revealed similar results [25]. However, whilst informative in nature, neither review conducted a quality appraisal, leaving an information gap about the specific methodological flaws of the current evidence.

To our knowledge, no previous review has exclusively assessed quantitative evidence focusing on the association between young caring and mental health. This review has three aims: (i) to assess differences in mental health outcomes between young carers and non-carers, (ii) to evaluate the international evidence on this association using a rigorous tool for quality assessment, and (iii) to identify the current methodological limitations that need to be addressed to clearly establish a causal link between exposure to young informal caring and subsequent mental health outcomes. Addressing these limitations with future research will potentially provide a better framework for policies and interventions aimed at improving the mental health outcomes of young informal carers.

## Methods

This systematic review was registered in PROSPERO (CRD42021251666) and follows the PRISMA guidelines for reporting systematic reviews (see Online Resource 1) [33]. The questions guiding this review are:

- Do mental health outcomes differ by informal caring status of people aged 25 years or less?
- Does the current quantitative literature of the association between young informal caring and mental health provide sufficient evidence to establish whether young informal care could lead to poor mental health?
- What are the methodological limitations that should be addressed in order to evaluate the impact of young informal caring on later mental health?

### Search strategy and eligibility criteria

A search strategy was developed in Medline (PubMED) in two steps. As a first step, a two-tiered search strategy searching title and abstract was used combining terms for “Young carers” and “Mental health” (see Table 1 for specific terms). As a second step, a three-tiered search strategy combining terms for “youth”, “caregivers” and “mental health” was developed. These were all combined in the final search. A complete list of search terms for each database can be found in Online Resource 2. Literature searches were conducted on four electronic databases including Medline (OVID), EMBASE (OVID), PsycInfo (OVID) and Web of Science on the 17th of March 2021. References of included studies were screened, and search alerts were set for each database to identify additional studies.

**Table 1:** Search strategy

STEP 1	
Tier 1	Tier 2
Terms related to young carers	Terms related to mental health
Young carer* or young caregiver* or caregiving youth or young caregiver* or young adult carer* or young adult caregiver* or young adult caregiver* or child carer* or child caregiver* or child care giver*	Mental health or psychological* or depressi* or depression or anxiety or anxiety disorders or Stress Disorders, Post-Traumatic or Posttraumatic Stress Disorder or Post traumatic Stress Disorder or Post-traumatic Stress Disorder or Stress, Psychological or Stress or Psychological Stress

STEP 2		
Tier 1*	Tier 2*	Tier 3
Terms related to caregiver	Terms related to youth	Mental health MeSH terms
Caregiver*	Young adult or Adolescent or Child	Mental Health/or Depression/or Anxiety disorders/or Stress Disorders, Traumatic/

^*^Tier 1 and 2 were restricted to titles

We formulated a list of eligibility criteria for inclusion of studies. Articles were included if they were of quantitative design and compared mental health outcomes between young carers and non-carers. Given our focus on synthesising quantitative evidence, studies with a prospective cohort, case–control, retrospective, cross-sectional, or intervention trial design were considered for inclusion. We excluded qualitative studies, reviews, protocols, theoretical papers and case reports. No restrictions were placed on publication year and country. We included studies with carers aged 25 years old or younger. Articles were excluded if they were focused on formal carers, carers older than 25 years of age or written in non-English language. Articles with hospital or institutional based sampling were also excluded due to their limited generalisability to the broader population. When studies using the same dataset were identified the least recent was excluded.

Our focus was on overall mental health outcomes including depressive symptoms, anxiety, post-traumatic stress disorder, psychological distress and any other form of mental health measure that could reflect the psychological impact of informal caring through stress related pathways. For inclusion, mental health outcomes could be measured: (i) by using validated measures of self-reported mental health symptomology, (ii) through health records, (iii) through diagnosis by a mental health professional, or (iv) by participants’ report of previously diagnosed mental illness.

### Study selection

Citations of search results were exported to Covidence, a web-based tool to conduct systematic reviews [34]. Two reviewers (LFA and JE) independently screened articles (title/abstract and full text) for inclusion. Both reviewers were blinded to each other’s decisions and disagreements were resolved through discussion. When disagreements could not be solved, a third reviewer (TK) was consulted.

### Data extraction and quality assessment

A data extraction form was constructed in Excel to include information about authors, year of publication, population focus, study design, sample size, characterisation of exposure, characterisation of the outcome and measures of effect. This form was piloted on 10% of the studies and adapted when necessary. Data extraction was conducted by one reviewer (LFA) and cross-checked by a second reviewer (JE). Authors of included studies were contacted to provide any missing data.

The quality of included studies was assessed using the Risk of Bias tool for non-randomised studies of exposures (ROBINS-E). ROBINS-E was preferred over other tools because it does not follow a study-design-based approach, and it was specifically tailored for observational studies on exposures [35, 36].

ROBINS-E evaluates non-randomised evidence based on their comparisons to the ideal target trial in three steps. We applied this as follows:

I. First, the reviewers defined a research question, minimal set of confounding factors and the accuracy of exposure and outcome measures that would be expected from the target trial. This was done by one reviewer (LFA), in consultation with other members of the team (TK, AS and GD).
II. Second, two reviewers (LFA and JE) independently described each individual study in the context of a hypothetical target trial.
III. Third, two reviewers (LFA and JE) independently assessed the risk of bias for each included study across seven domains (confounding, selection of participants into the study, classification of exposures, departure from intended exposures, missing data, measurement of outcomes and selection of the reported result). Risk of bias (RoB) was categorised as low, moderate, serious, and critical.

Overall RoB for each study was then recorded as the highest RoB for any domain. Item-level judgement for each domain of bias was recorded as the most dominant RoB.

Key findings of included studies were summarised in a descriptive table and discussed using a narrative synthesis. A meta-analysis was unfeasible for two reasons; first because none of the included studies were identified to be a low to moderate RoB, and second, due to the variability of outcome measures.

### Role of the founding source

The funders had no role in study design, data collection, data analysis, data interpretation, or writing of the report.

## Results

### Study characteristics

**Fig. 1:**
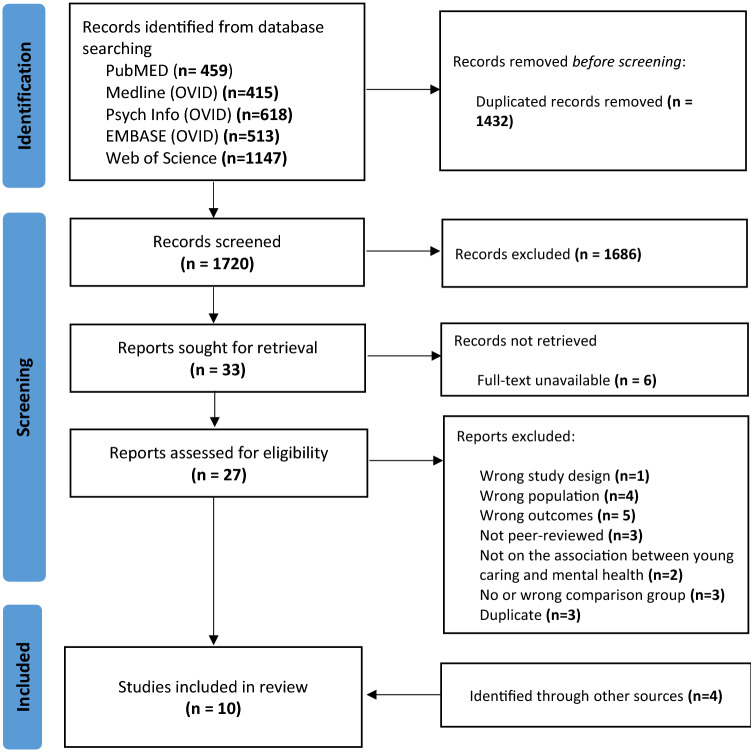
PRISMA flow diagram

We identified 3152 studies through database searching. After removing 1432 duplicates, a total of 1720 titles and abstract were screened. From these, 27 full-text articles were retrieved to assess eligibility. Of those, 21 were excluded because they did not meet the eligibility criteria. One additional article was found through screening authors’ bibliography and three through our search alerts. A total of 10 studies were therefore included in the review. The PRISMA diagram (Fig. 1) displays the selection process. The list of studies excluded as part of the full-text review, with reasons for exclusion, can be found in Online Resource 3.

All included studies were conducted in high income countries. The majority of studies were cross-sectional [28–31, 37–39], and only three used a longitudinal design [27, 40, 41]. Six studies looked at exposure to young caring [27, 28, 30, 31, 38, 41], while three assessed the extent of caring [29, 37] and one on substantial caregiving [39]. Mental health outcomes included depression [27–29, 31, 38], anxiety [28, 29, 38], other mental health/emotional problems [30, 37, 40, 41] and mental well-being [39, 41]. Mental health outcomes were self-reported using validated surveys for most studies [27–29, 31, 38–41], and the remaining two were based on a self-reported single-item measure of mental/emotional condition [30, 37]. A descriptive summary of included studies can be found in Table 2.

**Table 2:** Descriptive summary of included studies and main findings

First author (publication year)	Country	Study design	Analytical sample	*Exposure* and measurement	Mental health outcomes	Main findings
Nakanishi (2022)	UK	Longitudinal	3927	*Caregiving status* Participants were classified as informal carers if they provided support to someone ill, with a disability or an elderly person within or outside their households. Participants were asked to exclude paid caregiving	Mental health was measured using the Kessler Distress Scale (K6) Mental well-being was measured with the Warwick–Edinburgh Mental Well-being Scale	Psychological distress was higher among caregivers during the first wave of the COVID-19 pandemic, with a change of 0.60 (95%CI 0.07, 1.13) in K6 scores Mental well-being was lower among young carers with a − 0.30 (95%CI − 0.67, 0.08) difference in Warwick–Edinburgh Mental Well-being scores as compared to non-carers
Wepf (2021)	Sweden	Cross-sectional	2525	*Caregiving status and family health problems* Young carers were categorised as such if a person close to them “needed support due to an illness, mental health problems, disabilities, old age, addiction or injuries” and provided substantial support with domestic, personal, emotional or administrative tasks in the past six months. Non-carers were classified into non-carers with and without family health problems	Mental well-being was measured using the Warwick–Edinburgh Mental Well-being Scale	Non-caring was associated with higher levels of well-being, with an increase of 0.75 among non-carers with family health problems and a 1.75 increase on non-carers with healthy families, with young carers as the reference group
King (2021)	Australia	Longitudinal	2165	*Caregiving status and extent of caring* Participants were categorised as caregivers if they provided help to someone with a long-term condition, a disability or an elderly in the past six months. Volunteer activities and paid work were excluded. Caring status was categorised as carers and non-carers. Caregiving extent was categorised as no caring, less than daily caring and daily caring	Mental health status was measured using the Kessler Psychological Distress Scale (K10)	Providing informal care at ages 14/15 years was associated with greater psychological distress at 17/18 years, with an average treatment effect of 1.10 (95%CI 0.37, 1.83). A dose–response relationship was evident. When compared to non-carers, adolescents caring daily displayed an increase of 1.94 in Kessler Psychological Distress scores (95%: 0.48, 3.39), while those caring less than daily showed a 0.83 change (95%CI:0.06, 1.61)
Brimblecombe (2020)	UK	Longitudinal	All carers^a^: 6866, new carers^b^: 4067 young adults (16–25 years old)	*Caregiving status* Participants were categorised as young carers if they reported providing care for someone sick, with a disability, or elderly family member within or outside the household. Formal carers (paid and voluntary) were excluded. New carers were defined as those who started caring in 2014/2016	Mental health status was measured using the Mental Health Component Score (MCS) from the Short-form 12 Health Survey	Young carers, in all carers group, had poorer mental health, with a difference of − 2.75 in mean MCS (95%CI − 4.32, − 1.17) as compared to non-carers Among new carers, the mental health effects (ATE) of young caregiving were small, with a MCS coefficient of − 1.47 (95%CI − 3.77, 0.83)
Robison (2020)	Scotland	Cross-sectional	11,215 adolescents and young adults (11–18 years old)	*Caregiving status* Young carers were identified by asking participants if someone in their family household had a disability, long-term illness, drug/alcohol problem or mental health problem and if they looked after or cared for this person	Self-reported mental health problems were assessed by asking pupils whether they had a mental health/emotional illness	Young carers were more likely to report mental health problems (OR:1.35, 95%CI 1.11, 1.64) than non-carers
Haughland (2020)	Norway	Cross-sectional	40,205 young adults (18–25 years old)	*Caregiving status and daily hours of care* Caring status was defined as providing regular care for someone with physical or mental illness, disabilities, or substance misuse. Caring recipient could be a parent, sibling, another relative or a friend Hours of care: defined as hours spent caring for someone (not their child/children) in a regular weekday and weekend/holidays	Symptoms of anxiety and depression were assessed using the Hopkins Symptoms checklist (HSCL-25)	Young women carers were more likely to present w**i**th symptoms of anxiety and depression than non-carers in a dose–response manner, with ORs of 1.75 (95%CI:unavailable) for woman providing 1 or less hours of care and 2.47 (95%CI:unavailable) for those who reported 2 or more hours of daily care. Similar findings were reported for men providing 1 or less hours (OR: 1.71, 95%CI:unavailable) and 2 or more hours of care (OR:2.38, 95%CI:unavailable) Additional analyses showed small to moderate effect sizes of young caring on anxiety and depression for both men and women (ES:0.33 for 1 h or less and ES = 0.54 for 2 h or more)
Lakman (2019)	Canada	Cross-sectional	248 children, adolescents, and young adults (8–18 + years)	*Unclear* Young caregivers attending community agencies	A modified version of the Centre for Epidemiological Studies-Depression Scale (CES-D) was used to assess depressive symptoms Social anxiety: was measured using an adapted version of the Social Anxiety Scale for children-Revised	Effect sizes (Cohen’s d) for depression were large (ES:0.74), with young carers reporting a higher frequency of depressive symptoms (t (100): 3.68, *p* < 0.001) There were very small differences in social anxiety levels, F (3, 244): 1.95, *p*: 0.122, Wilk’s Λ: 0.97, partial η^2^:0.03
Tseliou (2018)	Northern Ireland	Cross-sectional	433,328 children, adolescents, and young adults (5–24 years)	*Caregiving status and weekly hours of care* Participants were asked if they provided care for someone with a long-term condition (physical, mental ill-health or disability) or problems related to old age. Responses categories included non-caregiver, caregiving for 1–19 h per week, 20–49 h per week, ≥ 50 h per week	Participants were classified as having chronic mental health problem if reporting an *“emotional, psychological or mental health condition (such as depression or schizophrenia)”*	Associations between young caregiving and chronic mental health differed by age. For younger participants (5–17 years old), caregiving was associated to increased odds of chronic mental health problems with a clear dose–response gradient, OR: 1.98 (95%CI 1.51, 2.59) for those caring 1–19 h per week and OR: 2.46 (95%CI 1.70, 3.56) for those caring for 20 h or more. Odds of chronic mental health problems in young adult carers were also increased with ORs of 1.34 (95%CI 1.17, 1.53) and 1.37 (95%CI 1.17, 1.61) for those caring 1–19 h and over 20 h, respectively
Greene (2017)	US	Cross-sectional	353 young adults (18–24 years)	*Caregiving status* Participants were asked if they were “providing assistance to a person who needs special medical care as a result of an injury, ageing, illness, disability, or other health condition”	Depression was measured using the Centre for Epidemiological Studies-Depression Scale (CES-D) Anxiety was assessed using the State Trait Anxiety Inventory (STAI)	Moderate effect sizes for the association between young caregiving and depression were reported (ES = 0.45) The effect size was also moderate for state anxiety (ES = 0.44) and small for trait anxiety (ES = 0.35)
Pakenham (2006)	Australia	Cross-sectional	245 children, adolescents, and young adults (10–24 years)	*Caregiving status* Participants were asked if they had parent with an illness/disability	Depression and anxiety were assessed using the Brief Symptom Inventory (BSI-18)	Carers and non-carers did not differ on depression and anxiety scores (measure of effect not reported)

^a^All carers at the start of follow-up

^b^Restricted to non-carers at the beginning of follow-up

### Risk of bias assessment

Table 3 displays results for the RoB assessment. The overall quality of evidence was low, with only one study rated at low risk [40] and the remaining rated at serious (*n* = 4) [27, 29, 30, 37, 39, 41] to critical (*n* = 3) risk of bias [28, 31, 38]. RoB was particularly high for the confounding domain where three studies were judged at critical risk [28, 31, 38], four at serious [27, 29, 37, 41] and two at moderate risk [30, 39] and one at low risk [40]. RoB for the selection of participants was mostly moderate (*n* = 4) [29, 30, 37, 39], with four studies rating at low [27, 37, 40, 41], one at serious [31] and the remaining one at critical risk [28]. Eight studies were rated at moderate RoB in the classification of exposures [27, 29, 30, 37–41], the remaining two were at serious [31] and critical risk [28]. Most studies were rated at low risk of bias in departures from intended exposures (*n* = 8), while the remaining two were rated at moderate risk [40, 41]. Only one study rated at serious risk of bias due to missing data [27] eight were judged at low risk [28–31, 37–40] and one at moderate risk [41]. The RoB judgement for outcome measurement was serious (*n* = 5) [28, 30, 31, 37, 39], mainly due to the risk of differential misclassification due to participant’s knowledge of their exposure status, the remaining papers rated at low (*n* = 3) [27, 29, 38] to moderate risk [40, 41]. RoB due to selection of the reported result was low for almost all studies, except for one which was rated at serious risk [31].

**Table 3:**
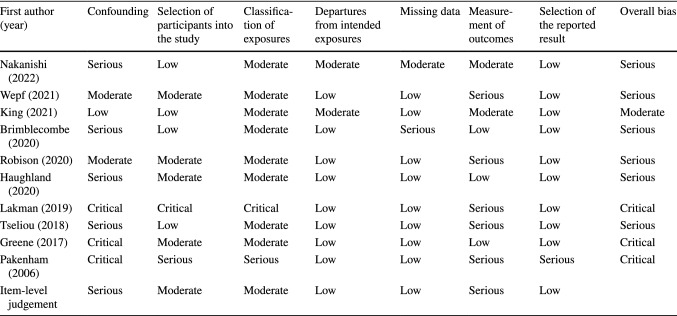
Risk of bias of non-randomised studies of exposures (ROBINS-E)

The main findings with effect sizes and 95% confidence intervals for all studies with available data are summarised in Table 2. Our qualitative synthesis of evidence focuses on those papers identified at serious [27, 29, 30, 37, 39, 41] and moderate risk [40], while those at critical RoB were excluded from our synthesis as recommended by Sterne et al. 2016 [42].

### Qualitative synthesis

Of the seven studies that were rated at moderate to serious RoB, four assessed young caring status [27, 30, 39, 41] and the remaining three assessed the extent of caregiving activities [29, 37, 40]. Five studies included a sample of children and adolescents [30, 37, 39–41], and five included young adults [27, 29, 30, 37, 39].

Drawing on longitudinal data from the UK Millennium Cohort Study, Nakanishi et al. [41] investigated the mental health effects of informal care among adolescents during the COVID-19 pandemic. Young carers could be providing support to someone in or outside their households, and any formal care was excluded. The authors found that compared to non-carers, adolescent carers were more likely to experience higher psychological distress, with a change of 0.60 (95%CI 0.07, 1.13) on total Kessler Distress scores (K6).

Wepf and Leu [39] examined mental well-being differences between young carers (aged 17–21 years) and their non-caring peers using cross-sectional data from schools and vocational training institutes in German-speaking Switzerland. Participants were classified as young carers if they provided substantial support with everyday activities to someone close. Authors compared mental health differences between young carers and non-carers with and without health problems in their families. Mental well-being was substantially better among non-carers without family health problems (mean difference: 1.72) than young carers. Differences in mental well-being were smaller when comparing non-carers with family health problems with young carers, with a difference of 0.75 on the Warwick–Edinburgh Mental Well-being scores.

King et al. [40] investigated the mental health effects of informal care among adolescents drawing on data from the Longitudinal Study of Australian Children. Informal carers could be providing support to anyone with a disability, long-term health condition or an older adult. Caring activities related to paid work and volunteering were excluded. Compared to non-carers, informal carers were more likely to display higher psychological distress scores 4 years later, with an average treatment effect (ATE) of 1.08 (95%CI 0.20, 1.81) on the Kessler Distress Scale (K10). Further analysis examined the extent of caregiving, showing a clear dose–response relationship. Compared to non-carers, daily caregivers showed an increase of 1.81 (95%CI 0.33, 3.28) in K10 4 years later, while the effect was smaller for non-daily carers (ATE: 0.83; 95%CI 0.33, 3.28).

Using cross-sectional data from the Glasgow city schools health and well-being survey, Robison et al. [30] looked at young people who were providing support to a family household member. Authors assessed the association between young caring status and the presence of mental health/emotional illness in students aged 11–18 years old. In the fully adjusted regression model, young carers were 35% more likely to report a mental or emotional illness than non-carers (95%CI 1.11, 1.64).

Brimblecombe et al. [27] examined mental health effects of young caring using longitudinal data from the UK Household Survey (UKHS) in people aged 16–25 years. Caring recipients included any household and non-household members who were receiving support from the young respondent, excluding volunteering activities or other forms of formal/semiformal caregiving. Mental health was measured using the mental health component score (MCS). Fully adjusted linear regression analysis showed that young carers had poorer mental health than non-carers of the same age (change in MCS: − 2.75; 95%CI − 4.32, − 1.17). Additional analysis using propensity score matching (PSM) and restricted to new carers showed that the mental health effects of young caring were small (change in MCS: − 1.47; 95%CI − 3.77, 0.83). Authors attributed the attenuated effects in the matched analysis to the fact that the analysis likely missed the long-term mental health effects of persistent caring roles.

Haugland et al. [29] investigated symptoms of depression and anxiety in people aged 18 to 25 years, drawing on cross-sectional data from the SHoT 2018 study, a national sample of Norwegian higher education students. Young carers were classified as those who provided help or support to a parent, sibling, another relative or someone else, excluding their own children. Fully adjusted regression analyses, stratified by gender, showed that young carers (women and men) were more likely to present symptoms of anxiety and depression than non-carers, and that these associations appeared in a dose–response manner. Anxiety and depression were 1.47 times more likely in woman caring for one hour or less per day and 2.47 times in those caring for 2 h or more per day as compared with women not providing care. Similarly, odds of depression were 1.71 increased in men providing up to one hour of care a day and 2.38 increased in those caring for more than two hours as compared to non-carers. Additional analyses for all carers showed small to moderate effects of young caring on scores for anxiety and depression.

Finally, Tseliou et al. [37] examined the association between weekly hours of care and participants’ reports of chronic mental health problems using 2011 census data from Northern Ireland. Analysis were stratified by age groups and young carers were classified as non-intense and intense carers according to weekly hours of care. Young carers could be providing support to a family member, friend, neighbour, or someone else, excluding care provision that was part of paid employment. Fully adjusted regression analysis showed that young carers were at higher odds of having poor chronic mental health than non-carers in both age groups. However, there was evidence of a dose response relationship only among very young carers (5–17 years old) with an odds ratio of 1.98 (95%CI 1.51, 2.59) for those caring 1–19 h per week and 2.46 (95%CI 1.70, 3.56) for those caring for 20 h or more.

## Discussion

This study presents the first systematic review of quantitative studies comparing mental health between young carers and their non-caring peers. Only three of the included studies used longitudinal data. The overall quality of evidence from this review was low: only one out of ten included studies was rated at moderate RoB and none at low risk. The sources of bias were mostly related to confounding and outcome measurement. Seven studies with the lowest risk of bias (moderate to serious) were included in the qualitative synthesis. Out of these, four were focused on young caring status and the other three on the duration of caring roles. Across the included studies, being a young carer was consistently associated with poorer mental health but information to support a causal effect was limited.

These findings are consistent with previous reviews that have found that young carers often experience feelings and symptoms of depression and anxiety [25, 26, 32]. The findings are also consistent with qualitative studies that have shown that the mental health of young carers is negatively affected by their caregiving activities [11, 19, 43–45], especially among those with a high burden of care [11]. Furthermore, these results align with studies among adult informal carers that have demonstrated that substantial caring demands lead to negative mental health outcomes [46].

Poor mental health outcomes in young carers may be related to isolation, stigma, and reduced time to participate in leisure activities [11, 45]. It is known that caring responsibilities can limit opportunities for social interaction and many young carers feel isolated from their peers. This sense of isolation could often be reinforced by their perception of dissonance from their friends and fear of embarrassment. When leisure activities are reported, these are usually limited to household-based activities or accompanied by domestic tasks, such as cleaning and cooking. This further isolates young carers, as the stigma related to their caring roles or their relative’s condition may prevent them from engaging in typical social activities such as inviting friends to their house [11].

An additional explanation for young carers poorer mental health pertains to their ongoing worry and concern for the health and well-being of their caring recipients [16, 21, 43]. Some young carers report living in a state of constant readiness due to the unpredictable nature of their relative’s condition [16]. Moreover, the psychological pressures of their caring roles may be intensified by a lack of preparation and understanding of the medical conditions they are required to deal with [16].

While the majority of included studies consistently reported a mental health penalty of being a young carer [27–30, 37, 38], one study (at critical risk of bias) reported no mental health differences between carers and non-carers [31]. It is possible that this contradictory result is related to the ascertainment of the exposure, where only those who reported having a parent with a disability were classified as young carers. Although most care recipients are the young carers’ parents, these roles are not restricted to only parental care [15]. Young carers also provide caring support to siblings and grandparents. Thus, ignoring this group could lead to misclassification of caring status (in which carers are classified as non-carers), and attenuate the observed mental health effects.

Consistent with previous reviews [25, 26, 32], this systematic review has highlighted the dearth of quantitative evidence of the associations between young caring and mental health, as well as some key methodological limitations in the current literature. In most studies reviewed, the definition of young carers was focused on caring status. The studies that examined the extent of caring found considerable mental health differences between those providing significant hours of care compared to those providing no care at all [29, 37, 40]. While the identification of young carers itself is problematic and broad definitions are usually preferred, these definitions can mask the mental health impact of caring [25]. This is because young people who engage in minor caring tasks are not substantially different from those who do not identify themselves as young informal carers [47]. Therefore, binary distinctions between carers and non-carers may be overly simplistic. To address this limitation, it is recommended that future research identifies and utilises more comprehensive ways of capturing the extent of caring activities, as well as the type of support provided by young people.

Furthermore, the vast majority of questions pertaining to young caring status in the reviewed studies focussed solely on disability or chronic conditions as reasons for caring, excluding substance or alcohol abuse. This is a noteworthy limitation as it is estimated that around 27% of young carers provide regular support to someone with issues related to alcohol or substance abuse [48]. Moreover, the mental health burden of caring for someone with these issues may be greater on young carers, as their caring responsibilities are likely less predictable and involve more emotional support [14]. Also, the stigma associated with alcohol and substance misuse might place them at risk of bullying victimisation and social withdrawal [14].

As previously mentioned, the available evidence for this review was largely cross-sectional, limiting our understanding of the causal effect of young caring on mental health. The temporal order in which the exposure (e.g. to informal caring) precedes mental health outcomes is an essential criterion for causal inference [49]. Longitudinal evidence is needed to assess whether caring leads to poor mental health among young carers, or whether poor mental health due to other factors, precedes young informal caring. Another major limitation was a lack of theoretical basis for confounding adjustment. This restricted the exchangeability of exposed and unexposed groups, another important criterion for contemporary causal approaches [49].

As a final point, we note that information on causal pathways from caring to mental health is limited. Understanding these pathways is imperative to inform key levers for intervening to improve the outcomes of young carers. It is also important to acknowledge that many young carers report benefits from these roles [11, 50–53]. Many report strong family bonds [51], resilience [53], and increased maturity [50]. They also report their caregiving status fosters empathy, compassion and a desire to help others [11]. Our immediate goal, therefore, should be focused on addressing the mechanisms through which caring leads to poor mental health and reducing the harms related to young caring, whilst also acknowledging potential benefits to the individual, as well as their significant contribution to our welfare systems.

This review has some limitations. First, due to variations in outcome measures and the low quality of the evidence overall, a meta-analysis was not feasible. Second, it is possible that some relevant papers were missed. Although we trialled search terms substantially, there is considerable variation in the way caring is expressed and operationalised across different cultural contexts. Therefore, our findings may be more applicable to contexts in which: (i) Informal caring is not normally expected from children, adolescents, and young adults, (ii) their identification aligns with international definitions and naming conventions of young carers as specified by Leu and Becker [1], and (iii) English-speaking countries.

This systematic review has highlighted an overall paucity of quantitative research with high methodological quality examining whether mental health outcomes differ by caring status in young people. Of the studies examined here, there was evidence that young carers do experience poorer mental health than their peers. This suggests that research attention should be directed to better understanding the key causal mechanisms underpinning these associations such that appropriate support and resources can be identified to optimise the mental health of young carers.

## Supplementary Information

Below is the link to the electronic supplementary material.

Supplementary file1 (PDF 130 KB)

Supplementary file2 (PDF 115 KB)

Supplementary file3 (PDF 145 KB)

## Data Availability

The data used in this systematic review are publicly available. The data extraction form and data used for the risk of bias assessment are available upon request.
