# Correction to: Mental health of young informal carers: a systematic review

**DOI:** 10.1007/s00127-022-02370-3

**Published:** 2022-10-01

**Authors:** Ludmila Fleitas Alfonzo, Ankur Singh, George Disney, Jennifer Ervin, Tania King

**Affiliations:** 1 Disability and Health Unit, Centre for Health Equity, Melbourne School of Population and Global Health, The University of Melbourne, Level 4, 207, Bouverie Street, Parkville, 3010 Australia; 2 Centre of Epidemiology and Biostatistics, Melbourne School of Population and Global Health, The University of Melbourne, Parkville, Victoria, Australia

## Correction to Social Psychiatry and Psychiatric Epidemiology 10.1007/s00127-022-02333-8

In the original article, Table 2 incorrectly states that the study “Wepf (2021)” was conducted in Sweden. The correct country classification of this study should read as Switzerland.

