# Supplementary material for: Mental health of young informal carers: a systematic review
Source: Soc Psychiatry Psychiatr Epidemiol. 2022 Jul 7;57(12):2345–58. doi: 10.1007/s00127-022-02333-8 (PMC9263065; doi:10.1007/s00127-022-02333-8)
Supplement: Supplementary file 2 — Supplementary file2 (PDF 115 KB) [file 127_2022_2333_MOESM2_ESM.pdf]

Mental health of young informal carers - a systematic review

Ludmila Fleitas Alfonzo[1], Ankur Singh[2], George Disney[1], Jennifer Ervin[1], Tania King[1]

Journal: Social Psychiatry and Psychiatric Epidemiology

Authors' affiliations:

[1] Centre for Health Equity, Melbourne School of Population and Global Health, The University of Melbourne, Parkville 3010, Australia.
[2] Centre of Epidemiology and Biostatistics, Melbourne School of Population and Global Health. The University of Melbourne, Parkville. Victoria, Australia.

Corresponding Author:
Ludmila Fleitas Alfonzo
E: ludmila.fleitasalfonzo@unimelb.edu.au

Online Resource 2. Search strategy

Medline (PubMed)

Young carer (Exposure)	Health (Outcome)
"Young carer*" [TIAB] OR "young caregiver*" [TIAB] OR "caregiving youth" [TIAB] OR "young caregiver*" [TIAB] OR "young adult carer*"[TIAB] OR "young adult caregiver*" [TIAB] OR "young adult caregiver*" [TIAB] OR "child carer*" [TIAB] OR "child caregiver*" [TIAB] OR "child care giver*" [TIAB]<br><br>OR<br><br>(Caregiver* [TI] AND ("Young adult" [TI] OR Adolescent [TI] OR Child [TI]))	"Mental health"[MH] OR "Mental health"[TIAB] OR psychological*[TIAB] OR depressi*[TIAB] OR depression[MH] OR anxiety[TIAB] OR "anxiety disorders"[MH] OR "Stress Disorders, Post-Traumatic" [MH] OR "Posttraumatic Stress Disorder" [TIAB] OR "Post traumatic Stress Disorder" [TIAB] OR "Post-traumatic Stress Disorder" [TIAB] OR "Stress, Psychological" [MH] OR Stress [TIAB] OR "Psychological Stress" [TIAB]

Searched on 17/3/2021: 459 results.

Medline (OVID)

1 (Young carer* or young caregiver* or caregiving youth or young caregiver* or young adult carer* or young adult caregiver* or young adult caregiver* or child carer* or child caregiver* or child care giver*).tw.
2 (Caregiver*).ti
3 ("Young adult" or Adolescent or Child).tw
4 2 and 3
5 1 or 4
6 (Mental health or psychological* or depressi* or depression or anxiety or anxiety disorders or Stress Disorders, Post-Traumatic or Posttraumatic Stress Disorder or Post traumatic Stress Disorder or Post-traumatic Stress Disorder or Stress, Psychological or Stress or Psychological Stress).tw.
7 Mental Health/ or Depression/ or Anxiety disorders/ or Stress Disorders, Traumatic/
8 6 or 7
9 5 or 8

Searched on 17/03/2021: 415 results.

Psych Info (OVID)

1 (Young carer* or young caregiver* or caregiving youth or young caregiver* or young adult carer* or young adult caregiver* or young adult caregiver* or child carer* or child caregiver* or child care giver*).tw.
2 (Caregiver*).ti

3 (“Young adult” or Adolescent or Child).ti.
4 2 and 3
5 1 or 4
6 (Mental health or psychological* or depressi* or depression or anxiety or anxiety disorders or Stress Disorders, Post-Traumatic or Posttraumatic Stress Disorder or Post traumatic Stress Disorder or Post-traumatic Stress Disorder or Stress, Psychological or Stress or Psychological Stress).tw.
7 Mental Health/ or Depression/ or Anxiety disorders/ or Stress Disorders, Traumatic/
8 6 or 7
9 5 or 8

Searched on 17/03/2021: 618 results.

EMBASE (OVID)

1 (Young carer* or young caregiver* or caregiving youth or young caregiver* or young adult carer* or young adult caregiver* or young adult caregiver* or child carer* or child caregiver* or child care giver*).tw.
2 (Caregiver*).ti.
3 (Young adult or Adolescent or Child).ti.
4 2 and 3
5 1 or 4
6 (Mental health or psychological* or depressi* or depression or anxiety or anxiety disorders or Stress Disorders, Post-Traumatic or Posttraumatic Stress Disorder or Post traumatic Stress Disorder or Post-traumatic Stress Disorder or Stress, Psychological or Stress or Psychological Stress).tw.
7 Mental Health/ or Depression/ or Anxiety disorders/ or Stress Disorders, Traumatic/
8 6 or 7
9 5 or 8

Searched on 17/3/2021: 513 results.

Web of science (Social Science citation Index)

1 TI = (“Young carer*” or “young caregiver*” or “caregiving youth” or “young caregiver*” or “young adult carer*” or “young adult caregiver*” or “young adult caregiver*” or “child carer*” or “child caregiver*” or “child care giver*”)
2 AB = (“Young carer*” or “young caregiver*” or “caregiving youth” or “young caregiver*” or “young adult carer*” or “young adult caregiver*” or “young adult caregiver*” or “child carer*” or “child caregiver*” or “child care giver*”)
3 TI = ((Caregiver*) AND (“Young adult” or Adolescent or Child))
4 TS= ("Mental health" or depression or "anxiety disorders" or "traumatic stress disorders")
5 TI = ("Mental health" or psychological* or depressi* or depression or anxiety or "anxiety disorders" or "Stress Disorders, Post-Traumatic" or "Posttraumatic Stress Disorder" or "Post traumatic Stress Disorder" or "Post-traumatic Stress Disorder or Stress, Psychological" or Stress or "Psychological Stress")
6 AB = ("Mental health" or psychological* or depressi* or depression or anxiety or "anxiety disorders" or "Stress Disorders, Post-Traumatic" or "Posttraumatic Stress Disorder" or "Post traumatic Stress Disorder" or "Post-traumatic Stress Disorder or Stress, Psychological" or Stress or "Psychological Stress")
7 #3 OR #2 OR #1
8 #6 OR #5 OR #4
9 #8 AND #7

Searched on 17/3/2021: 1147 results.
