# Supplementary material for: Mental health of young informal carers: a systematic review
Source: Soc Psychiatry Psychiatr Epidemiol. 2022 Jul 7;57(12):2345–58. doi: 10.1007/s00127-022-02333-8 (PMC9263065; doi:10.1007/s00127-022-02333-8)
Supplement: Supplementary file 3 — Supplementary file3 (PDF 145 KB) [file 127_2022_2333_MOESM3_ESM.pdf]

Mental health of young informal carers - a systematic review

Ludmila Fleitas Alfonzo[1], Ankur Singh[2], George Disney[1], Jennifer Ervin[1], Tania King[1]

Journal: Social Psychiatry and Psychiatric Epidemiology

Authors' affiliations:

[1] Centre for Health Equity, Melbourne School of Population and Global Health, The University of Melbourne, Parkville 3010, Australia.
[2] Centre of Epidemiology and Biostatistics, Melbourne School of Population and Global Health. The University of Melbourne, Parkville. Victoria, Australia.

Corresponding Author:
Ludmila Fleitas Alfonzo
E: ludmila.fleitasalfonzo@unimelb.edu.au

Online Resource 3. Articles excluded after full-text review with reasons for exclusion

	**Title**	**Reasons**
1	Dugan E. Book Review: How Caregiving Affects Development: Psychological Implications for Child, Adolescent, and Adult Caregivers. Rehabilitation Counseling Bulletin. 2011;54(4):236.	Wrong study design
2	Cohen SA, Cook S, Kelley L, Sando T, Bell AE. Psychosocial factors of caregiver burden in child caregivers: results from the new national study of caregiving. Health Qual Life Outcomes. 2015;13:120.	Wrong population
3	Rafnsson SB, Shankar A, Steptoe A. Informal caregiving transitions, subjective well-being and depressed mood: Findings from the English Longitudinal Study of Ageing. Aging Ment Health. 2017;21(1):104-12.	Wrong population
4	Strauss JR. Caregiving for parents and in-laws: commonalities and differences. J Gerontol Soc Work. 2013;56(1):49-66.	Wrong population
5	Coe NB, Van Houtven CH. Caring for mom and neglecting yourself? The health effects of caring for an elderly parent. Health Econ. 2009;18(9):991-1010.	Wrong population
6	Redmond G, Huynh J, Maurici V. How Big is the Gap in Wellbeing between Marginalised and Non-Marginalised Young People as They Approach Adolescence? Evidence from a National Survey of 9–14 Year Old Australians. Child Indicators Research. 2016;11(2):459-85.	Wrong outcomes
7	Hoyt MA, Mazza MC, Ahmad Z, Darabos K, Applebaum AJ. Sleep Quality in Young Adult Informal Caregivers: Understanding Psychological and Biological Processes. Int J Behav Med. 2021;28(1):6-13.	Wrong outcomes
8	Siskowski C. Young caregivers: effect of family health situations on school performance. J Sch Nurs. 2006;22(3):163-9.	Wrong outcomes
9	Diaz N, Siskowski C, Connors L. Latino Young Caregivers in the United States: Who are they and what are the Academic Implications of this Role? Child & Youth Care Forum. 2007;36(4):131-40.	Wrong outcomes
10	Pakenham KI, Cox S. The effects of parental illness and other ill family members on youth caregiving experiences. Psychol Health. 2015;30(7):857-78.	Wrong outcomes
11	Hibbert M. Health status of early adolescent non-caregivers and caregivers of a family member. Dissertation Abstracts International: Section B: The Sciences and Engineering. 2011;72(4-B):2032.	Not peer-reviewed

12	Deloney CG. Psychological and social varaibles impacting young adults caring for severely mentally ill mothers. Dissertation Abstracts International: Section B: The Sciences and Engineering. 2016;77(2-B(E)):No-Specified.	Not peer-reviewed
13	Sharer M. An ecological approach to the understanding of social support and mental health among South African children affected by AIDS. Dissertation Abstracts International: Section B: The Sciences and Engineering. 2016;76(10-B(E)):No-Specified.	Not peer-reviewed
14	Shifren K, Chong A. Health-Related Behaviors: A Study Among Former Young Caregivers. Journal of Adult Development. 2012;19(2):111-21.	Not on the association between young caregiving and mental health
15	Van der Werf HM, Luttik MLA, Francke AL, Roodbol PF, Paans W. Students growing up with a chronically ill family member; a survey on experienced consequences, background characteristics, and risk factors. Bmc Public Health. 2019;19(1):1486.	Not on the association between young caregiving and mental health
16	Becker S, Sempik J. Young Adult Carers: The Impact of Caring on Health and Education. Children & Society. 2018;33(4):377-86.	No or wrong comparison group
17	Cassidy T, Giles M, McLaughlin M. Benefit finding and resilience in child caregivers. Br J Health Psychol. 2014;19(3):606-18.	No or wrong comparison group
18	Järkestig-Berggren U, Bergman AS, Eriksson M, Priebe G. Young carers in Sweden—A pilot study of care activities, view of caring, and psychological well-being. Child & Family Social Work. 2018;24(2):292-300.	No or wrong comparison group
19	Bacharz KC, Goodmon LB. Abstracts of the 13th Annual Conference of the American Psychosocial Oncology Society, 3-5 March 2016, San Diego, California. Psychooncology. 2016;25 Suppl 2(SUPPL. 2):1-155.	Full-text unavailable
20	Hawken TA, Turner-Cobb J, Barnett J. Pathways to psychophysiological resilience in young informal caregivers: Mapping the impacts of chronic stress. Abstracts of the 77th Annual Scientific Meeting, March 6–9, 2019. Vancouver, BC, Canada. Psychosomatic Medicine. 2019;81(4):A1-A213.	Full-text unavailable
21	Hawken TA, Turner-Cobb J, Barnett J. An examination of hair cortisol in young caregivers: Social support and resilience. Psychoneuroendocrinology. 2019;107(Supplement):55.	Full-text unavailable
22	Holguin G, Ceullar R. Academic achievement and depressive-like symptoms among adolescent caregivers for grandparents with and without dementia. Annals of Behavioral Medicine. 2017;51:S1169-S70.	Full-text unavailable
23	Shu BC, Lung FW, Chen YC, Chen BC. Mental health in family caregivers with children with intellectual disability who receive a home care programme. Journal of Intellectual Disability Research. 2000;44:463-.	Full-text unavailable
24	Tseliou F, Rosato M, Maguire A, Wright D, O'Reilly D. Differential effect of caregiving across age-groups: A census-based record linkage study. The Lancet. 2017;390(SPEC.ISS 1):S90.	Full-text unavailable
25	Shifren K, Chong A. Health-Related Behaviors: A Study Among Former Young Caregivers. Journal of Adult Development. 2012;19(2):111-21.	Duplicate

26	Pakenham KI, Cox S. The effects of parental illness and other ill family members on youth caregiving experiences. Psychol Health. 2015;30(7):857-78.	Duplicate
27	Haugland BSM, Hysing M, Sivertsen B. The Burden of Care: A National Survey on the Prevalence, Demographic Characteristics and Health Problems Among Young Adult Carers Attending Higher Education in Norway. Frontiers in Psychology. 2020;10.	Duplicate
